# 3-Hy­droxy-2-phenyl-2,3,3a,7a-tetra­hydro-1*H*,5*H*-pyrano[3,2-*b*]pyrrol-5-one: crystal structure and Hirshfeld surface analysis

**DOI:** 10.1107/S2056989017005680

**Published:** 2017-04-21

**Authors:** Julio Zukerman-Schpector, Angélica V. Moro, Marcelo R. dos Santos, Carlos Roque D. Correia, Mukesh M. Jotani, Edward R. T. Tiekink

**Affiliations:** a Departmento de Química, Universidade Federal de São Carlos, 13565-905 São Carlos, SP, Brazil; b Instituto de Química, Universidade Estadual de Campinas, UNICAMP, CP 6154, 13084-971, Campinas, São Paulo, Brazil; c Instituto de Química, Universidade Federal do Rio Grande do Sul – UFRGS, CEP 91501-970 Porto Alegre, RS, Brazil; d Instituto de Ciências da Saúde, Universidade Paulista, CEP 70390-130, Brasília, DF, Brazil; e Department of Physics, Bhavan’s Sheth R. A. College of Science, Ahmedabad, Gujarat 380 001, India; f Research Centre for Chemical Crystallography, School of Science and Technology, Sunway University, 47500 Bandar Sunway, Selangor Darul Ehsan, Malaysia

**Keywords:** crystal structure, aza-isoaltholactone, hydrogen bonding, Hirshfeld surface analysis

## Abstract

The title isoaltholactone derivative, C_13_H_13_NO_3_, has an NH group in place of the ether-O atom in the five-membered ring of the natural product. The five-membered ring is twisted about the N—C bond linking it to the six-membered ring, which has a half-chair conformation with the O atom connected to the ether-O atom lying above the plane defined by the remaining atoms. The dihedral angle between the mean planes of the rings comprising the fused-ring system is 75.10 (8)°. In the crystal, hy­droxy-O—H⋯N(amine) hydrogen bonding sustains linear supra­molecular chains along the *a* axis. Chains are linked into a three-dimensional architecture *via* amine-N—H⋯π(phen­yl) and phenyl-C—H⋯O(hy­droxy) inter­actions. The influence of the amine-N—H⋯π(phen­yl) contact on the mol­ecular packing is revealed by an analysis of the Hirshfeld surface.

## Abstract 2

Twisted and half-chair conformations are found for the five- and six-membered rings comprising the fused-ring system in the title isoaltholactone derivative. In the mol­ecular packing, linear supra­molecular chains sustained by hy­droxy-O—H⋯N(amine) hydrogen bonding are evident.

## Chemical context   

Styryllactones are a diverse group of secondary metabolites which have demonstrated significant potency against a broad spectrum of human tumour cells, including breast, colon, kidney and pancreas cancer lines (Tian *et al.*, 2006 ▸). Other biological activities have also been revealed for this class of compound, namely anti-inflammatory, anti-microbial, anti-fertility and immunosuppressant (de Fatima *et al.*, 2006 ▸). A member of the styryllactone family of compounds is iso­altho­lactone, a natural product which comprises an α,β-unsaturated furan­opyran­one unit, *i.e*. there is an oxygen atom in place of the NH group in (I) shown in the Scheme. Iso­altho­lactone is structurally notable for its central tetra-substituted tetra­hydro­furan ring, which has four consecutive stereogenic centres. Compound (I), described herein, was originally prepared to enhance the biological activity of isoaltholactone (Moro *et al.*, 2011 ▸). Crystals of (I) have subsequently become available and the present report details the crystal and mol­ecular structures of (I) along with an analysis of the Hirshfeld surface of (I) in order to provide more information on the supra­molecular association.
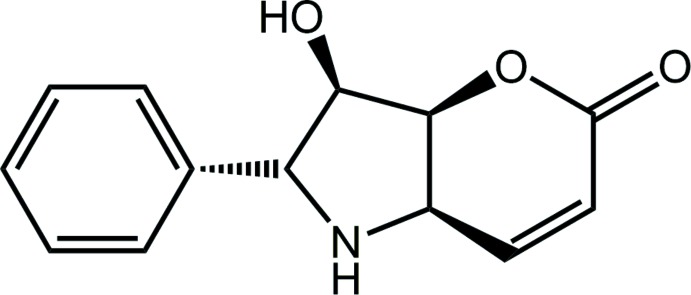


## Structural commentary   

**Figure 1:**
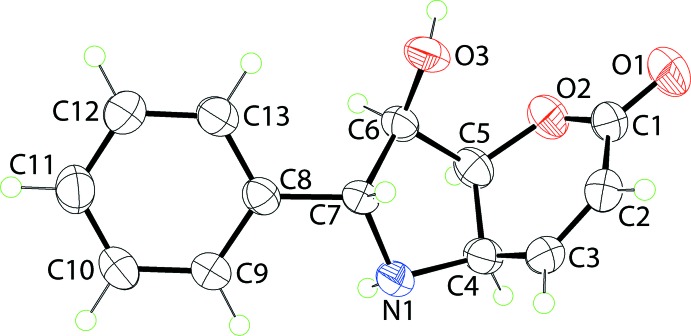
The mol­ecular structure of (I), showing the atom-labelling scheme and displacement ellipsoids at the 50% probability level.

The mol­ecular structure of (I) is shown in Fig. 1 ▸. The configurations about the chain of four chiral centres, *i.e*. C4–C7, are *R*, *S*, *R* and *R*, respectively. The five-membered pyrrolyl ring is twisted about the N1—C4 bond. The six-membered pyranyl ring is best described as having a half-chair conformation where the O1, C1–C4 atoms are co-planar (r.m.s. deviation = 0.0453 Å) and the C5 atom lies 0.435 (3) Å out of the plane. The fused-ring system has, to a first approximation, the shape of the letter V with the dihedral angle between the mean planes through each of the rings being 75.10 (8)°. The oxygen atoms all lie to one side of the plane through the pyrrolyl ring. Finally, the dihedral angle between the pyrrolyl and phenyl rings is 33.11 (7)°, indicating a twisted conformation.

## Supra­molecular features   

**Figure 2:**
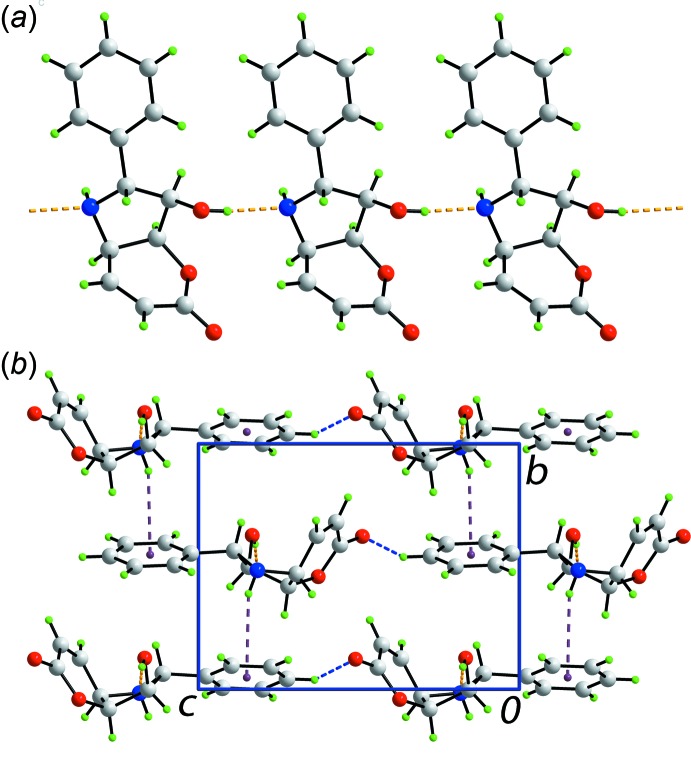
Mol­ecular packing in (I): (*a*) a view of the supra­molecular chain sustained by hy­droxy-O—H⋯N(amine) hydrogen bonding and (*b*) a view of the unit-cell contents shown in projection down the *a* axis. The O—H⋯N, N—H⋯π and C—H⋯O inter­actions are shown as orange, purple and blue dashed lines, respectively.

Conventional hy­droxy-O—H⋯N(amine) hydrogen bonding in the crystal of (I) leads to a linear, supra­molecular chain along the *a* axis as illustrated in Fig. 2 ▸ *a*, Table 1 ▸. The amine-N—H atom forms an inter­action with the phenyl ring, *i.e*. amine-N—H⋯π(phen­yl), Table 1 ▸, linking mol­ecules along the *c* axis, as shown in Fig. 2 ▸ *b*. The hy­droxy-O atom accepts a weak contact from a phenyl-H atom to connect mol­ecules along the *b* axis, thereby consolidating the three-dimensional mol­ecular packing (Fig. 2 ▸ *b*).

## Hirshfeld surface analysis   

**Figure 3:**
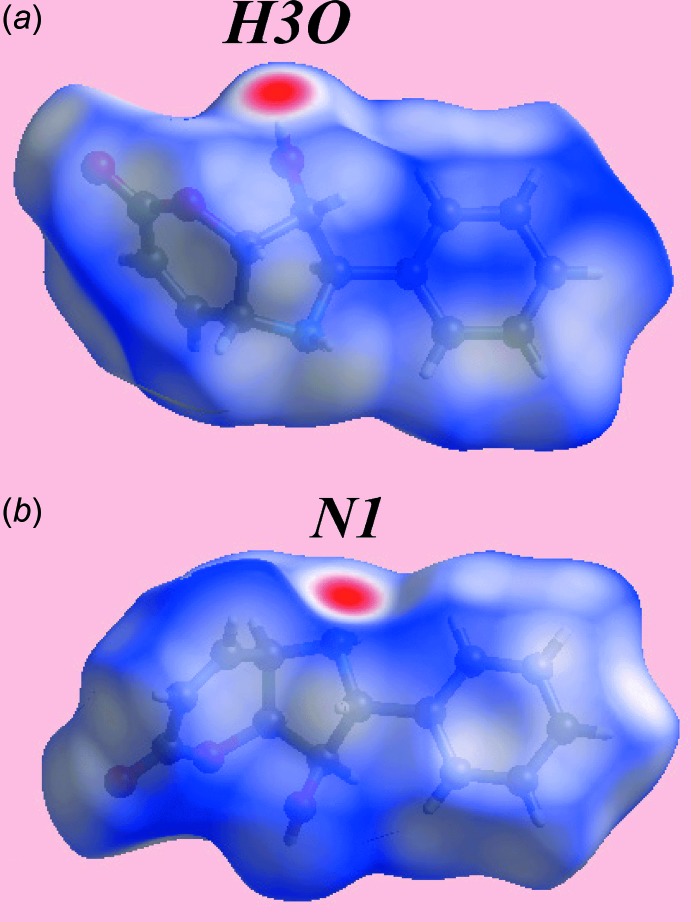
Two views of the Hirshfeld surface for (I) mapped over *d*_norm_ over the range −0.435 to 1.180 au.

**Figure 4:**
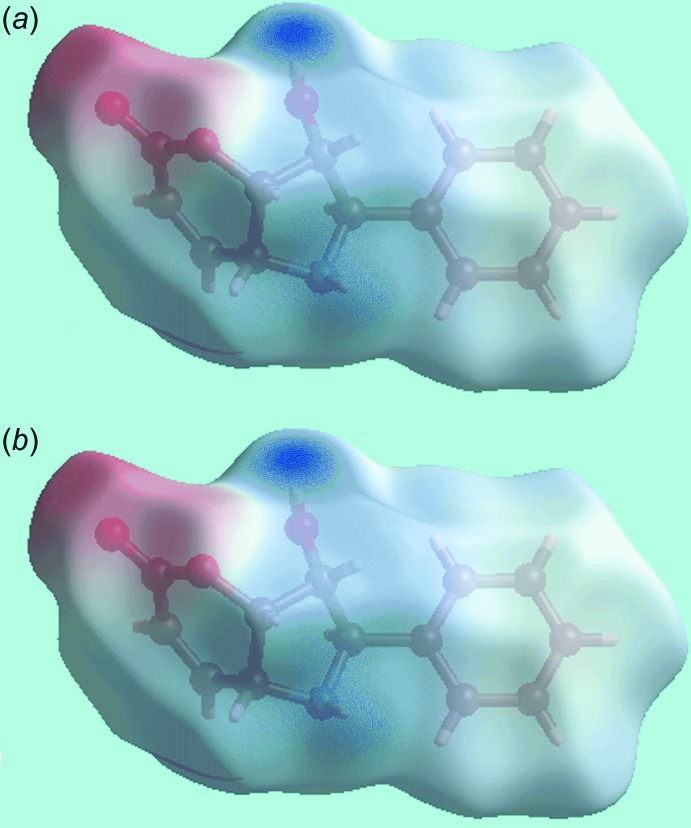
Two views of the Hirshfeld surfaces for (I) mapped over the calculated electrostatic potential over the range ±0.116 au. The red and blue regions represent negative and positive electrostatic potentials, respectively.

**Figure 5:**
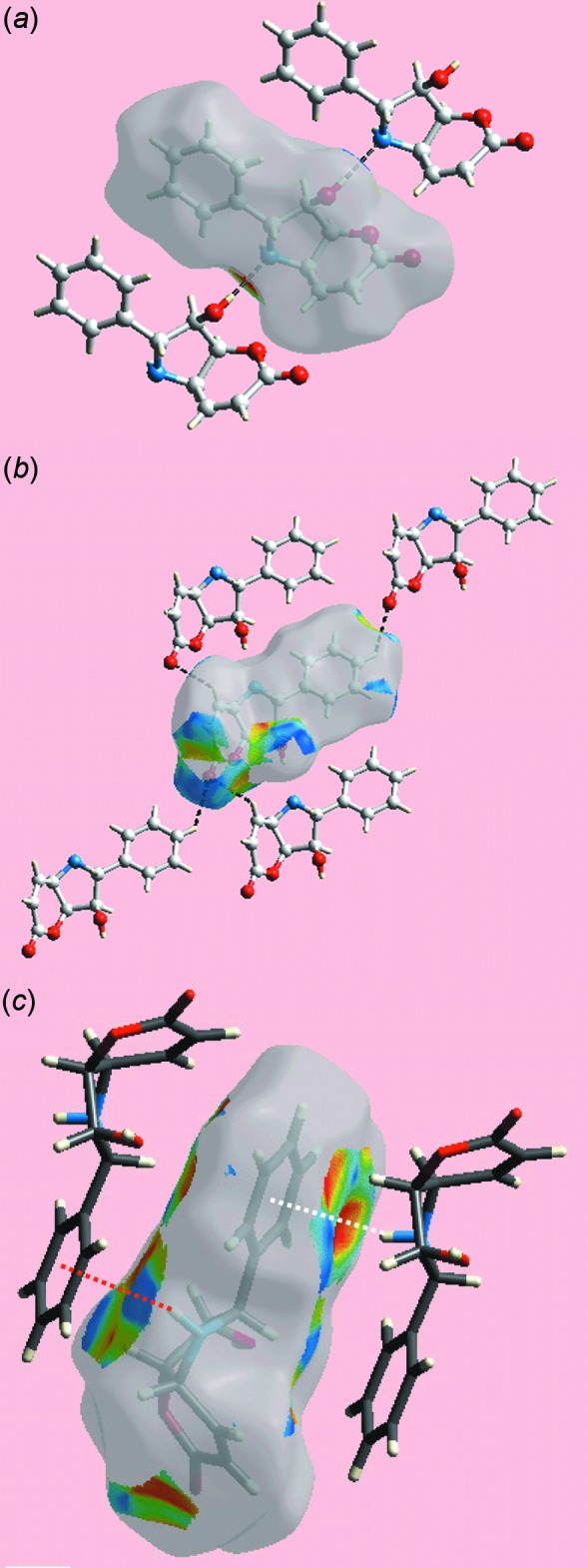
Views of Hirshfeld surface for a reference mol­ecule in (I) mapped over the shape-index property highlighting: (*a*) O—H⋯N hydrogen bonds (black dashed lines), (*b*) C—H⋯O inter­actions (black dashed lines) and (*c*) N—H⋯ π–π⋯H—N inter­actions as red- and white- dotted lines, respectively.

The Hirshfeld surfaces calculated for the structure of (I) provide additional insight into the supra­molecular association and was performed as per a recent publication (Wardell *et al.*, 2017 ▸). The appearance of bright-red spots at the hy­droxy-H3*O* and amine-N1 atoms on the Hirshfeld surfaces mapped over *d*_norm_ in Fig. 3 ▸ *a* and *b*, respectively, indicate the presence of conventional O—H⋯N hydrogen bonding leading to the linear supra­molecular shown in Fig. 2 ▸ *a*. The donor and acceptor atoms of this inter­action are also evident on the Hirshfeld surface mapped over the calculated electrostatic potential as blue (positive potential) and red regions (negative potential) near the respective atoms in Fig. 4 ▸. The presence of a blue region around the amine-H1*N* atom, Fig. 4 ▸ *a*, and a light-red region with a concave surface above the phenyl (C8–C13) ring, Fig. 4 ▸ *b*, are indicative of the N—H⋯π inter­action, shown to be influential on the packing. The immediate environments about a reference mol­ecule within shape-indexed-mapped Hirshfeld surface highlighting O—H⋯N hydrogen-bonding, weak inter­molecular C—H⋯O contacts and the N—H⋯π inter­action are illustrated in Fig. 5 ▸ *a*–*c*, respectively.

**Figure 6:**
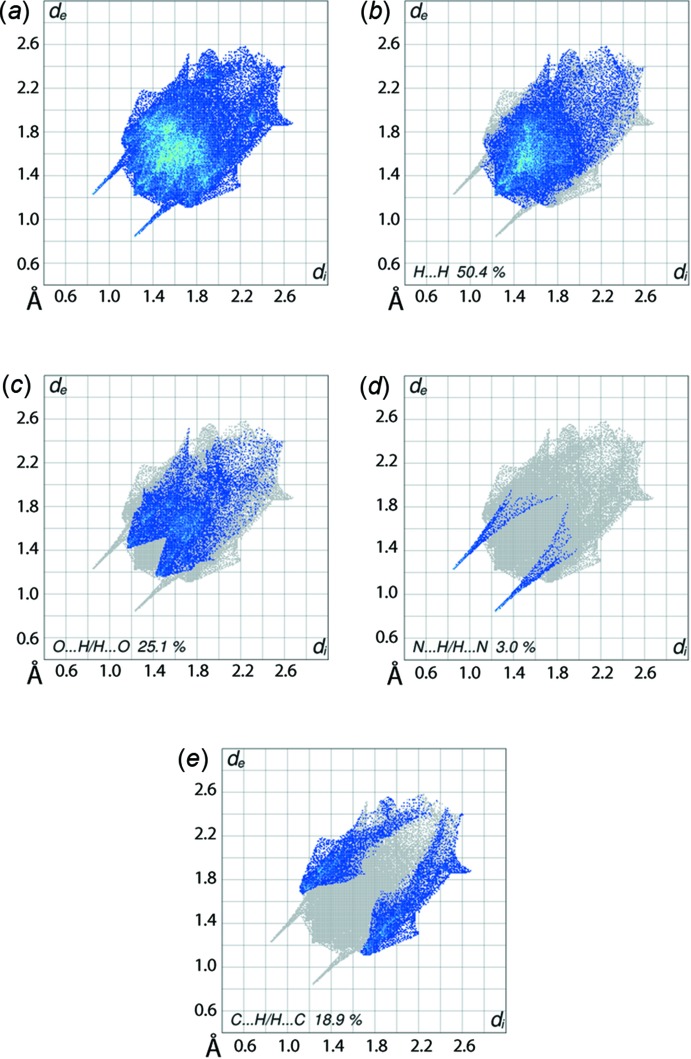
(*a*) The full two-dimensional fingerprint plots for (I) and fingerprint plots delineated into (*b*) H⋯H, (*c*) O⋯H/H⋯O, (*d*) N⋯H/H⋯H and (*e*) C⋯H/H⋯C contacts.

The overall two-dimensional fingerprint plot, Fig. 6 ▸ *a*, and those delineated into H⋯H, O⋯H/H⋯O, N⋯H/H⋯N and C⋯H/H⋯C contacts (McKinnon *et al.*, 2007 ▸) are illustrated in Fig. 6 ▸ *b*–*e*, respectively; the relative contributions from various contacts to the Hirshfeld surfaces are summarized in Table 2 ▸. It is clear from the fingerprint plot delineated into H⋯H contacts, Fig. 6 ▸ *b*, that in spite of contributing the maximum, *i.e*. 50.4%, to the Hirshfeld surface, these contacts do not have a significant influence upon the mol­ecular aggregation as the atoms are separated at distances greater than the sum of their van der Waals radii.

Despite the absence of characteristic faint-red spots expected on the *d*_norm_-mapped Hirshfeld surface for (I), Fig. 3 ▸, the two-dimensional fingerprint plot delineated into O⋯H/H⋯O contacts, Fig. 6 ▸ *c*, highlights the weak inter­molecular C—H⋯O contacts, Fig. 5 ▸ *b*. The distribution of points in the form of two adjoining cones with the peaks at *d*_e_ + *d*_i_ ∼ 2.6 Å confirms the presence of these contacts as well as the short inter-atomic O⋯H/H⋯O contacts listed in Table 3 ▸. A pair of well-separated spikes with the tips at *d*_e_ + *d*_i_  ∼ 2.1 Å in the fingerprint plot delineated into N⋯H/H⋯N contacts, Fig. 6 ▸ *d*, results from the presence of the O—H⋯N hydrogen bond. In the fingerprint plot delineated into C⋯H/H⋯C contacts, Fig. 6 ▸ *e*, these contacts appear as the distribution of points having a pair of peaks around *d*_e_ + *d*_i_ ∼ 2.8 Å. The short inter-atomic C⋯H/H⋯C contacts involving the amine-HN1, pyranyl-H5 and phenyl-carbon C10, C12 and C13 atoms, Table 3 ▸, arise from the presence of N—H⋯π(phen­yl) inter­actions. Their reciprocal, *i.e*. π⋯H—N inter­actions, are recognized from similar short inter-atomic contacts involving pyranyl-H7 and phenyl-carbon atoms C9 and C10, Fig. 5 ▸ *c* and Table 3 ▸. The small contribution of 1.3% from O⋯O and C⋯O/O⋯C contacts exert a negligible influence on the packing.

## Database survey   

**Figure 7:**
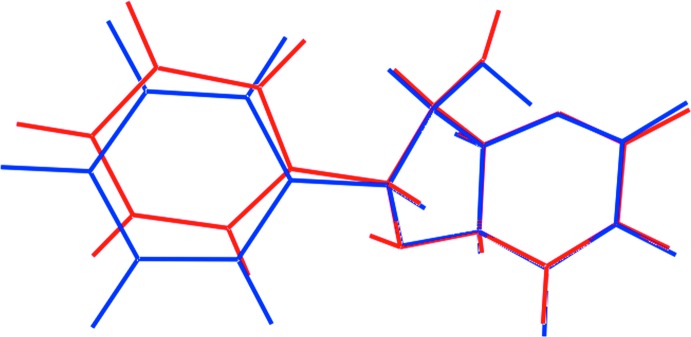
Mol­ecular overlay diagram of (I) and (+)-isoaltholactone shown as red and blue images, respectively.

As mentioned in the *Chemical context*, compound (I) is an aza derivative of the biologically active species (+)-isoaltholactone whereby the ether-oxygen atom of the five-membered ring of the latter has been substituted with a NH group. Indeed, the structure of (+)-isoaltholactone (Colegate *et al.*, 1990 ▸) is the most closely related structure to (I) in the crystallographic literature (Groom *et al.*, 2016 ▸). A structural overlay diagram of (I) and (+)-isoaltholactone is shown in Fig. 7 ▸ from which it can be seen the conformations exhibit a high degree of agreement, the only difference relating to the relative orientations of the terminal phenyl group. The mol­ecular framework of (I) comprising the two fused-rings linked by a C*sp* ^3^—C*sp* ^3^ single bond is without precedent in the crystallographic literature. However, there are two examples where the link between the five- and six-membered rings is a double bond, namely 3-acetyl-2-methyl­isochromeno[4,3-*b*]pyrrol-5(1*H*)-one (Pathak *et al.*, 2011 ▸) and 8-methyl­isochromeno[4,3-*b*]indol-5(11*H*)-one (Meng *et al.*, 2014 ▸).

## Synthesis and crystallization   

The compound was prepared as described in the literature (Moro, *et al.*, 2011 ▸). Crystals for the present study were obtained by vapour diffusion of hexane into ethyl ether solution of (I).

## Refinement   

Crystal data, data collection and structure refinement details are summarized in Table 4 ▸. Carbon-bound H atoms were placed in calculated positions (C—H = 0.95–1.00 Å) and were included in the refinement in the riding-model approximation, with *U*_iso_(H) set to 1.2*U*_eq_(C). The O- and N-bound H atoms were located from a difference map, but refined with O—H = 0.84±0.01 Å and N—H = 0.88±0.01 Å, and with *U*_iso_(H) = 1.5*U*_eq_(O) and 1.2*U*_eq_(N). As the value of the Flack parameter was ambiguous, the absolute structure is based on that of the starting material employed in the reaction (Moro, *et al.*, 2011 ▸).

## Supplementary Material

Crystal structure: contains datablock(s) . DOI: 10.1107/S2056989017005680/hg5487sup1.cif

Structure factors: contains datablock(s) I. DOI: 10.1107/S2056989017005680/hg5487Isup2.hkl

Supporting information file. DOI: 10.1107/S2056989017005680/hg5487Isup3.cml

CCDC reference: 1543983

Additional supporting information:  crystallographic information; 3D view; checkCIF report

## Figures and Tables

**Table 1:** Hydrogen-bond geometry (Å, °) *Cg*1 is the centroid of the C8–C13 ring.

*D*—H⋯*A*	*D*—H	H⋯*A*	*D*⋯*A*	*D*—H⋯*A*
O3—H3*O*⋯N1^i^	0.86 (2)	2.07 (2)	2.920 (3)	174 (4)
N1—H1*N*⋯*Cg*3^ii^	0.87 (1)	2.88 (2)	3.705 (3)	160 (2)
C11—H11⋯O1^iii^	0.95	2.60	3.280 (3)	129

Symmetry codes: (i) 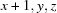; (ii) 
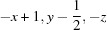
; (iii) 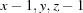.

**Table 2:** Percentage contributions of inter-atomic contacts to the Hirshfeld surface for (I)

Contact	percentage contribution
H⋯H	50.4
O⋯H/H⋯O	25.1
C⋯H/H⋯C	18.9
N⋯H/H⋯N	3.0
C⋯O/O⋯C	1.3
O⋯O	1.3

**Table 3:** Summary of short inter-atomic contacts (Å) in (I)

Contact	distance	symmetry operation
H1*N*⋯C12	2.888 (18)	1 − *x*, − 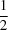 + *y*, −*z*
H1*N*⋯C13	2.875 (19)	1 − *x*, − 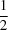 + *y*, −*z*
H5⋯C10	2.89	1 − *x*, − 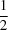 + *y*, −*z*
H7⋯C9	2.84	1 − *x*, 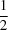 + *y*, −*z*
H7⋯C10	2.80	1 − *x*, 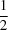 + *y*, −*z*
H2⋯O2	2.64	2 − *x*, 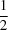 + *y*, 1 − *z*
H3⋯O1	2.62	−1 + *x*, *y*, *z*
C3⋯O1	3.209 (3)	−1 + *x*, *y*, *z*

**Table 4:** Experimental details

Crystal data	
Chemical formula	C_13_H_13_NO_3_
*M*_r_	231.24
Crystal system, space group	Monoclinic, *P*2_1_
Temperature (K)	100
*a*, *b*, *c* (Å)	5.9638 (2), 8.4266 (3), 11.0246 (4)
β (°)	92.779 (3)
*V* (Å^3^)	553.39 (3)
*Z*	2
Radiation type	Mo *K*α
μ (mm^−1^)	0.10
Crystal size (mm)	0.40 × 0.40 × 0.20

Data collection	
Diffractometer	Bruker SMART APEXII
Absorption correction	Multi-scan (*SADABS*; Sheldrick, 1996 ▸)
*T*_min_, *T*_max_	0.914, 1.000
No. of measured, independent and observed [*I* > 2σ(*I*)] reflections	4550, 2377, 2149
*R*_int_	0.017
(sin θ/λ)_max_ (Å^−1^)	0.650

Refinement	
*R*[*F* ^2^ > 2σ(*F* ^2^)], *wR*(*F* ^2^), *S*	0.036, 0.094, 1.03
No. of reflections	2377
No. of parameters	160
No. of restraints	3
H-atom treatment	H-atom parameters not refined
Δρ_max_, Δρ_min_ (e Å^−3^)	0.14, −0.19
Absolute structure	Flack *x* determined using 856 quotients [(*I* ^+^)−(*I* ^−^)]/[(*I* ^+^)+(*I* ^−^)] (Parsons *et al.*, 2013 ▸)
Absolute structure parameter	0.7 (5)

Computer programs: *APEX2* and *SAINT* (Bruker, 2007 ▸), *SHELXS97* (Sheldrick, 2008 ▸), *SHELXL2014* (Sheldrick, 2015 ▸), *ORTEP-3 for Windows* (Farrugia, 2012 ▸), *QMol* (Gans & Shalloway, 2001 ▸), *DIAMOND* (Brandenburg, 2006 ▸) and *publCIF* (Westrip, 2010 ▸).

## supplementary crystallographic information

### Crystal data

**Table:**

C_13_H_13_NO_3_	*F*(000) = 244
*M**_r_* = 231.24	*D*_x_ = 1.388 Mg m^−^^3^
Monoclinic, *P*2_1_	Mo *K*α radiation, λ = 0.71073 Å
*a* = 5.9638 (2) Å	Cell parameters from 2450 reflections
*b* = 8.4266 (3) Å	θ = 2.4–27.3°
*c* = 11.0246 (4) Å	µ = 0.10 mm^−^^1^
β = 92.779 (3)°	*T* = 100 K
*V* = 553.39 (3) Å^3^	Block, colourless
*Z* = 2	0.40 × 0.40 × 0.20 mm

### Data collection

**Table:**

Bruker SMART APEXII diffractometer	2377 independent reflections
Radiation source: sealed tube	2149 reflections with *I* > 2σ(*I*)
Graphite monochromator	*R*_int_ = 0.017
φ and ω scans	θ_max_ = 27.5°, θ_min_ = 1.9°
Absorption correction: multi-scan (SADABS; Sheldrick, 1996)	*h* = −6→7
*T*_min_ = 0.914, *T*_max_ = 1.000	*k* = −10→10
4550 measured reflections	*l* = −14→14

### Refinement

**Table:**

Refinement on *F*^2^	H-atom parameters not refined
Least-squares matrix: full	*w* = 1/[σ^2^(*F*_o_^2^) + (0.0534*P*)^2^ + 0.049*P*] where *P* = (*F*_o_^2^ + 2*F*_c_^2^)/3
*R*[*F*^2^ > 2σ(*F*^2^)] = 0.036	(Δ/σ)_max_ < 0.001
*wR*(*F*^2^) = 0.094	Δρ_max_ = 0.14 e Å^−^^3^
*S* = 1.03	Δρ_min_ = −0.19 e Å^−^^3^
2377 reflections	Absolute structure: Flack *x* determined using 856 quotients [(*I*^+^)-(*I*^-^)]/[(*I*^+^)+(*I*^-^)] (Parsons *et al.*, 2013)
160 parameters	Absolute structure parameter: 0.7 (5)
3 restraints	

### Special details

**Table:**

Geometry. All esds (except the esd in the dihedral angle between two l.s. planes) are estimated using the full covariance matrix. The cell esds are taken into account individually in the estimation of esds in distances, angles and torsion angles; correlations between esds in cell parameters are only used when they are defined by crystal symmetry. An approximate (isotropic) treatment of cell esds is used for estimating esds involving l.s. planes.

### Fractional atomic coordinates and isotropic or equivalent isotropic displacement parameters (Å^2^)

**Table:**

	*x*	*y*	*z*	*U*_iso_*/*U*_eq_	
O1	1.1372 (3)	0.1225 (4)	0.51075 (18)	0.0772 (7)	
O2	1.0038 (3)	−0.0413 (2)	0.37308 (15)	0.0519 (5)	
O3	1.0370 (3)	0.1267 (2)	0.16741 (16)	0.0474 (4)	
H3O	1.171 (3)	0.091 (5)	0.176 (3)	0.071*	
N1	0.4839 (3)	−0.0156 (3)	0.18328 (16)	0.0399 (4)	
H1N	0.463 (4)	−0.1113 (19)	0.156 (2)	0.048*	
C1	0.9838 (4)	0.0886 (4)	0.44096 (19)	0.0497 (6)	
C2	0.7749 (4)	0.1800 (3)	0.4292 (2)	0.0499 (6)	
H2	0.7692	0.2817	0.4661	0.060*	
C3	0.5947 (4)	0.1256 (4)	0.3691 (2)	0.0452 (5)	
H3	0.4612	0.1873	0.3664	0.054*	
C4	0.5952 (4)	−0.0302 (3)	0.3053 (2)	0.0424 (5)	
H4	0.5202	−0.1133	0.3538	0.051*	
C5	0.8350 (4)	−0.0795 (3)	0.2802 (2)	0.0427 (5)	
H5	0.8361	−0.1971	0.2683	0.051*	
C6	0.8844 (3)	−0.0009 (3)	0.15679 (18)	0.0371 (5)	
H6	0.9389	−0.0821	0.0990	0.044*	
C7	0.6538 (3)	0.0636 (3)	0.11123 (18)	0.0325 (4)	
H7	0.6510	0.1788	0.1330	0.039*	
C8	0.6046 (3)	0.0531 (3)	−0.02427 (19)	0.0343 (4)	
C9	0.3983 (4)	0.0059 (3)	−0.0742 (2)	0.0429 (5)	
H9	0.2824	−0.0227	−0.0222	0.051*	
C10	0.3576 (4)	−0.0004 (3)	−0.1992 (2)	0.0483 (6)	
H10	0.2153	−0.0342	−0.2319	0.058*	
C11	0.5227 (4)	0.0422 (3)	−0.2758 (2)	0.0495 (6)	
H11	0.4947	0.0384	−0.3614	0.059*	
C12	0.7296 (4)	0.0904 (4)	−0.2272 (2)	0.0513 (6)	
H12	0.8446	0.1197	−0.2796	0.062*	
C13	0.7697 (4)	0.0962 (3)	−0.1027 (2)	0.0457 (6)	
H13	0.9122	0.1302	−0.0703	0.055*	

### Atomic displacement parameters (Å^2^)

**Table:**

	*U*^11^	*U*^22^	*U*^33^	*U*^12^	*U*^13^	*U*^23^
O1	0.0479 (11)	0.130 (2)	0.0521 (10)	0.0000 (13)	−0.0175 (9)	−0.0153 (14)
O2	0.0411 (9)	0.0693 (12)	0.0437 (8)	0.0104 (8)	−0.0133 (7)	0.0077 (9)
O3	0.0286 (7)	0.0602 (10)	0.0528 (9)	−0.0071 (7)	−0.0035 (7)	0.0029 (9)
N1	0.0318 (9)	0.0502 (11)	0.0374 (9)	−0.0081 (9)	−0.0018 (7)	0.0015 (9)
C1	0.0378 (12)	0.0800 (19)	0.0307 (10)	−0.0039 (12)	−0.0044 (9)	0.0034 (12)
C2	0.0434 (13)	0.0707 (17)	0.0356 (11)	0.0002 (12)	0.0022 (10)	−0.0095 (11)
C3	0.0333 (11)	0.0673 (15)	0.0351 (10)	0.0030 (11)	0.0037 (8)	−0.0010 (11)
C4	0.0341 (11)	0.0546 (14)	0.0383 (11)	−0.0068 (10)	−0.0009 (8)	0.0079 (11)
C5	0.0398 (12)	0.0440 (12)	0.0433 (12)	0.0033 (10)	−0.0084 (10)	0.0047 (10)
C6	0.0278 (9)	0.0451 (12)	0.0379 (10)	0.0038 (9)	−0.0027 (8)	−0.0032 (10)
C7	0.0258 (9)	0.0366 (10)	0.0348 (10)	0.0011 (8)	−0.0010 (8)	0.0003 (9)
C8	0.0309 (10)	0.0354 (10)	0.0363 (10)	0.0033 (8)	−0.0025 (9)	0.0000 (8)
C9	0.0322 (10)	0.0561 (14)	0.0400 (11)	−0.0028 (10)	−0.0014 (9)	−0.0019 (11)
C10	0.0391 (11)	0.0620 (15)	0.0426 (12)	−0.0018 (12)	−0.0098 (10)	−0.0040 (12)
C11	0.0535 (14)	0.0582 (14)	0.0363 (11)	0.0033 (12)	−0.0036 (11)	0.0019 (11)
C12	0.0468 (13)	0.0663 (18)	0.0410 (12)	−0.0055 (12)	0.0039 (10)	0.0090 (12)
C13	0.0373 (12)	0.0560 (14)	0.0433 (12)	−0.0076 (10)	−0.0032 (9)	0.0050 (11)

### Geometric parameters (Å, º)

**Table:**

O1—C1	1.201 (3)	C5—H5	1.0000
O2—C1	1.334 (4)	C6—C7	1.540 (3)
O2—C5	1.437 (3)	C6—H6	1.0000
O3—C6	1.409 (3)	C7—C8	1.511 (3)
O3—H3O	0.852 (13)	C7—H7	1.0000
N1—C4	1.476 (3)	C8—C9	1.382 (3)
N1—C7	1.477 (3)	C8—C13	1.390 (3)
N1—H1N	0.869 (13)	C9—C10	1.388 (3)
C1—C2	1.465 (4)	C9—H9	0.9500
C2—C3	1.317 (3)	C10—C11	1.376 (4)
C2—H2	0.9500	C10—H10	0.9500
C3—C4	1.490 (4)	C11—C12	1.383 (4)
C3—H3	0.9500	C11—H11	0.9500
C4—C5	1.527 (3)	C12—C13	1.383 (3)
C4—H4	1.0000	C12—H12	0.9500
C5—C6	1.554 (3)	C13—H13	0.9500
			
C1—O2—C5	120.30 (18)	C7—C6—C5	103.38 (16)
C6—O3—H3O	110 (3)	O3—C6—H6	110.3
C4—N1—C7	103.78 (16)	C7—C6—H6	110.3
C4—N1—H1N	107.0 (17)	C5—C6—H6	110.3
C7—N1—H1N	108.7 (18)	N1—C7—C8	113.57 (17)
O1—C1—O2	117.9 (3)	N1—C7—C6	106.87 (16)
O1—C1—C2	123.3 (3)	C8—C7—C6	115.42 (17)
O2—C1—C2	118.7 (2)	N1—C7—H7	106.8
C3—C2—C1	122.1 (3)	C8—C7—H7	106.8
C3—C2—H2	119.0	C6—C7—H7	106.8
C1—C2—H2	119.0	C9—C8—C13	118.11 (19)
C2—C3—C4	121.6 (2)	C9—C8—C7	122.51 (19)
C2—C3—H3	119.2	C13—C8—C7	119.36 (19)
C4—C3—H3	119.2	C8—C9—C10	121.1 (2)
N1—C4—C3	110.2 (2)	C8—C9—H9	119.5
N1—C4—C5	103.95 (18)	C10—C9—H9	119.5
C3—C4—C5	110.40 (19)	C11—C10—C9	120.3 (2)
N1—C4—H4	110.7	C11—C10—H10	119.9
C3—C4—H4	110.7	C9—C10—H10	119.9
C5—C4—H4	110.7	C10—C11—C12	119.4 (2)
O2—C5—C4	116.09 (19)	C10—C11—H11	120.3
O2—C5—C6	111.85 (19)	C12—C11—H11	120.3
C4—C5—C6	105.18 (17)	C13—C12—C11	120.2 (2)
O2—C5—H5	107.8	C13—C12—H12	119.9
C4—C5—H5	107.8	C11—C12—H12	119.9
C6—C5—H5	107.8	C12—C13—C8	121.0 (2)
O3—C6—C7	108.71 (18)	C12—C13—H13	119.5
O3—C6—C5	113.67 (18)	C8—C13—H13	119.5
			
C5—O2—C1—O1	−174.2 (2)	C4—N1—C7—C8	164.56 (19)
C5—O2—C1—C2	7.4 (3)	C4—N1—C7—C6	36.1 (2)
O1—C1—C2—C3	−167.4 (3)	O3—C6—C7—N1	−137.08 (18)
O2—C1—C2—C3	10.9 (4)	C5—C6—C7—N1	−16.0 (2)
C1—C2—C3—C4	−2.3 (4)	O3—C6—C7—C8	95.6 (2)
C7—N1—C4—C3	76.7 (2)	C5—C6—C7—C8	−143.36 (19)
C7—N1—C4—C5	−41.6 (2)	N1—C7—C8—C9	13.6 (3)
C2—C3—C4—N1	−135.3 (2)	C6—C7—C8—C9	137.5 (2)
C2—C3—C4—C5	−21.0 (3)	N1—C7—C8—C13	−168.2 (2)
C1—O2—C5—C4	−32.2 (3)	C6—C7—C8—C13	−44.3 (3)
C1—O2—C5—C6	88.5 (3)	C13—C8—C9—C10	0.8 (4)
N1—C4—C5—O2	155.49 (19)	C7—C8—C9—C10	179.1 (2)
C3—C4—C5—O2	37.3 (3)	C8—C9—C10—C11	−0.7 (4)
N1—C4—C5—C6	31.3 (2)	C9—C10—C11—C12	0.3 (4)
C3—C4—C5—C6	−86.9 (2)	C10—C11—C12—C13	−0.2 (4)
O2—C5—C6—O3	−18.5 (3)	C11—C12—C13—C8	0.4 (4)
C4—C5—C6—O3	108.4 (2)	C9—C8—C13—C12	−0.7 (4)
O2—C5—C6—C7	−136.12 (18)	C7—C8—C13—C12	−179.0 (2)
C4—C5—C6—C7	−9.3 (2)		

### Hydrogen-bond geometry (Å, º)

Cg1 is the centroid of the C8–C13 ring.

**Table:**

*D*—H···*A*	*D*—H	H···*A*	*D*···*A*	*D*—H···*A*
O3—H3*O*···N1^i^	0.86 (2)	2.07 (2)	2.920 (3)	174 (4)
N1—H1*N*···*Cg*3^ii^	0.87 (1)	2.88 (2)	3.705 (3)	160 (2)
C11—H11···O1^iii^	0.95	2.60	3.280 (3)	129

Symmetry codes: (i) *x*+1, *y*, *z*; (ii) −*x*+1, *y*−1/2, −*z*; (iii) *x*−1, *y*, *z*−1.
